# Prevalence and clinical implications of atrial fibrillation in patients hospitalized due to COVID-19: Data from a registry in Poland

**DOI:** 10.3389/fcvm.2023.1133373

**Published:** 2023-03-13

**Authors:** Michał Terlecki, Wiktoria Wojciechowska, Marek Klocek, Tomasz Drożdż, Maryla Kocowska-Trytko, Paweł Lis, Christopher Pavlinec, Jan W. Pęksa, Michał Kania, Zbigniew Siudak, Andrzej Januszewicz, Reinhold Kreutz, Maciej Małecki, Tomasz Grodzicki, Marek Rajzer, CraCov HHS Investigators

**Affiliations:** ^1^ 1st Department of Cardiology, Interventional Electrocardiology and Arterial Hypertension, Jagiellonian University Medical College, Kraków, Poland; ^2^ Department of Metabolic Diseases and Diabetology, Jagiellonian University Medical College, Kraków, Poland; ^3^ Faculty of Medicine and Health Sciences, Jan Kochanowski University, Kielce, Poland; ^4^ Department of Hypertension, National Institute of Cardiology, Warsaw, Poland; ^5^ Charite-Universitatsmedizin Berlin, Corporate Member of Freie Universität Berlin, Humboldt-Universität zu Berlin, and Berlin Institute of Health, Institut für Klinische Pharmakologie und Toxikologie, Berlin, Germany; ^6^ Department of Internal Diseases and Geriatrics, Jagiellonian University Medical College, Kraków, Poland

**Keywords:** atrial fibrillation, COVID-19, prognosis, anticoagulation, NOAC drugs, MACE

## Abstract

**Background:**

Atrial fibrillation (AF) is a common arrhythmia with increasing prevalence with respect to age and comorbidities. AF may influence the prognosis in patients hospitalized with Coronavirus disease 2019 (COVID-19). We aimed to assess the prevalence of AF among patients hospitalized due to COVID-19 and the association of AF and in-hospital anticoagulation treatment with prognosis.

**Methods and results:**

We assessed the prevalence of AF among patients hospitalized due to COVID-19 and the association of AF and in-hospital anticoagulation treatment with prognosis. Data of all COVID-19 patients hospitalized in the University Hospital in Krakow, Poland, between March 2020 and April 2021, were analyzed. The following outcomes: short-term (30-days since hospital admission) and long-term (180-days after hospital discharge) mortality, major cardiovascular events (MACEs), pulmonary embolism, and need for red blood cells (RBCs) transfusion, as a surrogate for major bleeding events during hospital stay were assessed. Out of 4,998 hospitalized patients, 609 had AF (535 pre-existing and 74 *de novo*). Compared to those without AF, patients with AF were older and had more cardiovascular disorders. In adjusted analysis, AF was independently associated with an increased risk of short-term {*p* = 0.019, Hazard Ratio [(HR)] 1.236; 95% CI: 1.035–1.476} and long-term mortality (Log-rank *p* < 0.001) as compared to patients without AF. The use of novel oral anticoagulants (NOAC) in AF patients was associated with reduced short-term mortality (HR 0.14; 95% CI: 0.06–0.33, *p* < 0.001). Moreover, in AF patients, NOAC use was associated with a lower probability of MACEs (Odds Ratio 0.3; 95% CI: 0.10–0.89, *p* = 0.030) without increase of RBCs transfusion.

**Conclusions:**

AF increases short- and long-term risk of death in patients hospitalized due to COVID-19. However, the use of NOACs in this group may profoundly improve prognosis.

## Introduction

Since the beginning of the global pandemic, more than 620 million people worldwide have developed coronavirus disease 19 (COVID-19) and there were almost more than 6 million fatal cases globally (1). An infection with severe acute respiratory syndrome coronavirus 2 (SARS-CoV-2), at the peak of the pandemic, was one of the leading causes of death worldwide, with an overall death number comparable to those of cardiovascular disease (CVD) or cancer (1, 2). Although there is now a significant decrease in the number of cases globally and the clinical course of the infection appears to be somewhat milder than in the initial phases of the pandemic, it should be considered that SARS-CoV-2 will remain in our population permanently. Hence, in the public health domain, there is still a need for an analysis of the impact of chronic non-communicable diseases (NCDs) on clinical course of COVID-19. Moreover, there is an urgent need to evaluate the influence of COVID-19 on the effectiveness of therapeutic regimens used to date for the prevention of specific events among patients with NCDs including those with cardiovascular diseases.

Respiratory system involvement is still the most common clinical manifestation of the SARS-CoV-2 infection (3, 4). However, it has been shown that the cardiovascular system is also often affected and the combination of COVID-19 and preexisting CVD or cardiovascular risk factors results in a worse prognosis for both conditions (infection and CVDs) (3–8). Atrial fibrillation (AF) is the most common arrhythmia in the general population with increasing prevalence with age and/or the presence of coexisting comorbidities such as arterial hypertension, diabetes, heart failure, pulmonary disease (9, 10). Therefore, the impact of AF on the clinical course and prognosis of patients with COVID-19 seems important.

During the acute phase of COVID-19, especially in patients with a severe clinical course, factors such as, inflammation, hypoxia, dehydration and excessive sympathetic nervous system activation or even direct myocardial injury may promote the occurrence of *de novo* AF (5–12). In fact, in many observational retrospective studies the increased prevalence of AF among hospitalized COVID-19 patients was described (11–19).

So far, it has been already reported in some studies that patients hospitalized due to COVID-19 with concomitant arrhythmias, particularly with AF, have worse outcomes (13–18). However, the mechanism responsible for the increased risk of poor prognosis in patients with AF remains unclear. It appears possible that an increased inflammatory host response may be related to this relationship (14). However, there are no comprehensive data available about the impact of either previously diagnosed or *de novo* AF on the frequency rate of adverse events or short-term in-hospital prognosis and post-discharge mortality.

Since the start of the pandemic, multiple studies have demonstrated the link between COVID-19 and the risk for thrombotic events, both in the arterial and venous vascular bed (20–23). Several mechanisms responsible for this increased risk have been identified, i.e.,: the cytokine storm leading to hyperinflammation, endothelial dysfunction, platelet activation and coagulopathy (4, 24). There is no general consensus about the strategy for using anticoagulants including the prophylaxis of venous thromboembolism and an individualized approach in COVID-19 patients based on the severity of the infection has been suggested (21, 25, 26). Moreover, possible interactions between particularly oral anticoagulants and other drugs (e.g., antivirals, antibiotics or antiarrhythmics) should be taken into consideration (21). Nevertheless, in patients with AF and COVID-19, the same risk stratification according to the CHA_2_DS_2_-VASc score for the use of oral anticoagulation therapy as for the general population has been recommended (10).

The aim of our study was to evaluate the prevalence and impact of AF on mortality in COVID-19 patients and to assess the role of oral anticoagulation therapy on mortality, MACE and venous thromboembolic (pulmonary embolism) events among patients with AF that were hospitalized due to COVID-19.

## Materials and methods

### Study design

We retrospectively analyzed the medical records of all consecutive patients who were admitted due to a SARS-CoV2 infection to the University Hospital in Krakow between March 6, 2020 and April 30, 2021. Patients were diagnosed with COVID-19 according to the World Health Organization (WHO) and Polish guidelines with the use of RT-PCR method (rhino-oropharyngeal swab positivity for SARS CoV-2 RNA) (27–29). The treatment algorithm for COVID-19 was in accordance with the recommendations of the Polish Association of Epidemiologists and Infectiologists (27, 28). Individual patient data were obtained from the Hospital Information System. The presence of cardiovascular risk factors and CVD, other diagnoses and previous treatments were identified based on the medical history of patients and defined according to current European Society of Cardiology (ESC) guidelines (30).

Clinical data including demographics, medical history, inpatient clinical course, laboratory results, treatment, and in-hospital outcomes were obtained from the electronic medical records used by the University Hospital of Kraków. B.1.1.7 (Alfa) variant of SARS-CoV2 was the predominant strain of COVID-19 in Poland at the time we carried our study (31). Details of the analysis of the clinical course of COVID-19 among our patients according to the period/wave of the pandemic are presented in a separate publication (31). Preexisting AF was identified based on the previous medical history and documentation, while *de novo* AF was defined as an electrocardiogram (ECG) finding of AF during the hospital stay in a patient without a prior history of AF. In our study the term “AF” refers to both pre-existing and newly diagnosed, except where it has been clearly emphasized that we are discussing the pre-existing or newly diagnosed subgroups in which case we use the term pre-existing AF or *de novo* AF, respectively. The CHA_2_DS_2_-VASc score was calculated according to the current ESC guidelines (10). The estimated glomerular filtration rate (eGFR) was calculated from the Modification of Diet in Renal Disease (MDRD) formula (32).

The primary outcome in our study was the incidence of death from any cause on short-term (30-days since hospital admission) and long-term (180-days after hospital discharge) period. The last was assessed using data obtained from the National Electronic Population Registration System in Poland.

The secondary outcomes analyzed during hospitalization period were: major adverse cardiovascular events (MACEs) (stroke, myocardial infarction or acute limb ischemia), pulmonary embolism (as an equivalent of a venous thromboembolic event), the need for red blood cells (RBCs) transfusion (as a surrogate for major bleeding events).

The study was conducted in accordance with the guidelines of the Declaration of Helsinki and was approved by the Bioethics Committee of Jagiellonian University, decision number 1072.6120.278.2020.

### Statistical analysis

Categorical variables were presented as numbers and percentages. Continuous variables were expressed as mean and standard deviation (SD) or median and interquartile range (IQR). Normality was assessed by the Shapiro–Wilk test. We divided the study population into two groups according to presence of AF (patients with AF and patients without AF). Differences between groups were analyzed using the Student's or Welch's *t*-test depending on the equality of variances for normally distributed variables. The Mann–Whitney *U*-test was used for non-normally distributed continuous variables. Cox-proportional hazards models were fit to determine the adjusted associations between AF and primary and secondary outcomes. Factors influencing short-term mortality were identified in logistic regression model (33). Because age, male sex, heart failure, coronary artery disease, arterial hypertension, diabetes mellitus, chronic kidney disease (CKD) and chronic obstructive pulmonary disease (COPD) are established risk factors for poor outcome in COVID-19 patients (34), these variables were also included into the final model. Moreover, the severity of inflammation on admission (defined as increasing high-sensitivity C reactive protein [hsCRP] serum levels per 10 mmol/L were included in our Cox-proportional hazard analyses. In order to assess the impact of anticoagulation treatment on short-term mortality, a subgroup analysis with a Cox-proportional hazard model was performed in the cohort of patients with AF. In this analysis, we excluded individuals who required treatment with mechanical ventilation or renal replacement therapy. In addition, AF patients who were treated during hospital stay by at least two different anticoagulants or were switched from any anticoagulant agent to another (e.g., from vitamin K antagonists (VKA) to novel oral anticoagulants (NOAC), or low molecular weight heparin (LMWH) to VKA/NOAC or vice versa, respectively) were excluded. In this analysis, the same confounding factors were used as described above. Adjusted hazard ratios (HRs), along with 95% confidence intervals (CIs), were computed for all covariates. The proportional hazards model assumptions were checked using the Schoenfeld test and graphical diagnostics. Furthermore, to analyze long-term survival, Kaplan–Meier curves were-drawn for the group of patients who were discharged alive from hospital with a diagnosis of AF. In all analyses, a *p*-value of 0.05 or less was considered statistically significant. The statistical analysis was performed with the IBM SPSS 24.0 software package, STATA software, version 15 and R Core Team (2020).

## Results

### Study population and clinical characteristics

Overall, 4,998 patients (median [IQR] age 64.0 [52.0–74.0] years) were admitted to the University Hospital in Krakow due to COVID-19 and completed their hospital course (i.e., from admission to discharge or death). Our study population included 2,247 (45.0%) women and 2,751 (55.0%) men. There were a total of 609 (12.2%) patients with AF including 74 patients with *de novo* AF (1.5%) and 535 (10.7%) with a history of pre-existing AF. Demographic and clinical characteristics of the patients stratified by the presence of AF are shown in Table 1.

**Table 1:** Basic characteristics of participants.

Characteristics	Patients without AF *N* = 4,389 (87.8%)	Patients with AF *N* = 609 (12.2%)	*p*-value^a^
Age, years, median (IQR)	63.0 (49.0–72.0)	76.0 (69.0–83.0)	<0.001
Male sex, no. (%)	2,420 (55.1)	331 (54.4)	0.373
BMI^b^, kg/m^2^, mean (SD)	29.21 (5.79)	28.86 (5.67)	0.746
*Pre-Existing conditions, n* (%)			
Arterial hypertension, no. (%)	2,461 (56.1)	508 (83.4)	<0.001
Hyperlipidemia, no. (%)	835 (19.0)	213 (35.0)	<0.001
Diabetes Mellitus, no. (%)	1,099 (25.0)	241 (39.6)	<0.001
CAD, no. (%)	616 (14.0)	226 (37.1)	<0.001
History of MI, no. (%)	320 (7.3)	127 (20.9)	<0.001
Heart Failure, no. (%)	276 (6.3)	218 (35.8)	<0.001
AF *de novo*, no. (%)	n/a	74 (12.2)	<0.001
History of Stroke, no. (%)	281 (6.4)	103 (16.9)	<0.001
Asthma, no. (%)	261 (5.9)	41 (6.7)	0.247
COPD, no. (%)	213 (4.9)	55 (9.0)	<0.001
Chronic kidney disease, no. (%)	372 (8.5)	98 (16.1)	<0.001

AF, atrial fibrillation; BMI, body mass index; CAD, coronary artery disease; COPD, chronic obstructive pulmonary disease; MI, myocardial infarction; n/a, non-applicable, yrs, years.

Data are presented as mean (SD), median (Q1–Q3) or number (%).

^a^ For between AF group and no AF group difference.

^b^ Data available for 2,299 patients.

Patients with AF, in comparison to the rest of the cohort were older and were more likely to have the following comorbidities: arterial hypertension, diabetes, hypercholesterolemia, coronary artery disease, heart failure, chronic kidney disease, COPD, previous history of stroke and previous history of myocardial infarction (MI) (Table 1).

Clinical characteristics and drug therapy among patients with AF and without AF are presented in Table 2. Patients with AF had a lower systolic blood pressure (BP), and diastolic BP and higher respiratory rate at admission than patients without AF. There were no statistically significant differences between patients with AF and those without AF in respect to heart rate and oxygen saturation. Regarding biochemical parameters, patients with AF had higher N-terminal Prohormone of Brain Natriuretic Peptide (NTproBNP), high-sensitivity cardiac troponin (hs cTn), serum creatinine and lactate levels in comparison to patients without AF. There were no statistically significant differences in respect to hsCRP level between patients with AF and without AF. Patients with AF were more frequently treated with various cardiovascular drugs including angiotensin converting enzyme inhibitors (ACE-I), angiotensin II receptor blockers (ARB), beta-blockers, loop diuretics, statins, and antiplatelet drugs. AF patients were as expected more frequently treated with NOACs or VKA, but received less often LMWH as compared to patients without AF.

**Table 2:** Clinical characteristics and drug therapy among patients without AF and with AF.

Characteristics	Patients without AF	Patients with AF	*p*-value^a^
Number	*N* = 4,389 (87.8%)	*N* = 609 (12.2%)	
*Parameters on admission*			
SBP^b^, mmHg, median (IQR)	130.00 (118.00; 144.00)	129.00 (112.00; 143.00)	0.019
DBP^b^, mmHg, median (IQR)	80.00 (70.00; 89.00)	77.00 (68.00; 88.00)	<0.001
Heart Rate^b^/min, median (IQR)	84.00 (75.00; 95.00)	82.00 (72.00; 100.00)	0.823
Respiratory Rate^b^/min, median (IQR)	14.00 (12.00; 18.00)	16.00 (14.00; 20.00)	<0.001
Oxygen saturation^b^, %, median (IQR)	95.00 (92.00; 97.00)	95.00 (92.00; 97.00)	0.430
hsCRP^b^, mg/L, median (IQR)	51.40 (15.30; 104.00)	53.00 (19.93; 102.00)	0.169
D-dimer^b^, mg/L, median (IQR)	0.97 (0.55; 2.08)	0.94 (0.46; 2.19)	0.210
Procalcitonin, ng/ml, median (IQR)	0.08 (0.02; 0.21)	0.10 (0.04; 0.3)	<0.001
IL-6, pg/ml^b^, median (IQR)	31.64 (12.12; 73.36)	39.65 (15.99; 92.59)	0.002
NT-proBNP^b^, pg/ml, median (IQR)	367.00 (125.00; 1,350.00)	2213.50 (927.00; 5624.00)	<0.001
hs cTn, ng/ml, median (IQR)	8.78 (3.69; 28.11)	24.36 (10.09; 82.49)	<0.001
Creatinine, µmol/L, median (IQR)	76.90 (62.20; 98.60)	94.50 (72.90; 134.00)	<0.001
eGFR, ml/min/1,73 m^2^, median (IQR)	87.94 (76.58; 99.60)	113.82 (93.28; 148.80)	<0.001
Lactate, mmol/L^b^, median (IQR)	1.40 (1.00; 1.90)	1.60 (1.20; 2.10)	<0.001
*Cardiovascular therapy*			
Loop diuretics, no. (%)	1,226 (27.9)	307 (50.4)	<0.001
CCB, no. (%)	605 (13.8)	87 (14.3)	0.388
ACEI/ARB, no. (%)	865 (19.7)	144 (23.6)	0.014
Beta-blocker, no. (%)	1,155 (26.3)	325 (53.4)	<0.001
Antiplatelet drug, no. (%)^c^	641 (14.6)	110 (18.1)	0.016
Statin, no. (%)	559 (12.7)	154 (25.3)	<0.001
NOAC, no. (%)	128 (2.9)	184 (30.2)	<0.001
VKA, no. (%)	16 (0.4)	36 (5.9)	<0.001
Low Molecular Weight Heparin, no. (%)	2,481 (56.5)	250 (41.1)	<0.001
*Clinical course*			
ECMO, no. (%)	19 (0.4)	1 (0.2)	0.280
Mechanical ventilation, no. (%)	570 (13.0)	65 (10.7)	0.059
Renal replacement therapy, no. (%)	285 (6.5)	50 (8.2)	0.069
High flow oxygen therapy, no. (%)	575 (13.1)	81 (13.3)	0.466
Passive oxygen therapy, no. (%)^d^	1,423 (32.4)	197 (32.3)	0.506
Admission to an ICU, no. (%)	562 (12.8)	64 (10.5)	0.060
Catecholamine use, no. (%)	524 (1.9)	75 (12.3)	0.415

ACEI, angiotensin-converting enzyme (ACE) inhibitors; AF, atrial fibrillation; ARB, angiotensin II receptor blockers; CCB, calcium-channel blockers; DBP, diastolic blood pressure; ECMO, extracorporeal membrane oxygenation; eGFR, estimated glomerular filtration rate; hsCRP, high-sensitivity C-reactive protein; hs cTn, high-sensitivity cardiac troponin; ICU, intensive care unit; IL-6, interleukin 6; NOAC, novel oral anticoagulants; NTproBNP, N-terminal prohormone of brain natriuretic peptide; RBCs, packed red blood cells; SBP, systolic blood pressure; VKA, vitamin K antagonist.

Data are presented as mean (SD), median (Q1–Q3) or number (%).

^a^ For between AF group and No AF group difference;.

^b^ Data available in: 4,344 patients for SBP and DBP; 3,057 patients for NT-pro BNP; 3,820 patients for hs cTn - high-sensitivity cardiac troponin; 4,846 patients for procalcitonin; 2,909 patients for lactates; 4,833 patients for creatinine and eGFR; 4,641 patients for lymphocytes; 4,582 patients for D-dimer; 2,957 patients 2,957 patients for IL-6; 4,111 patients for Oxygen saturation; 4,415 patients for Heart rate; 3,607 patients for Respiratory rate;.

^c^ Antiplatelet = included those treated with low dose aspirin or/and clopidogrel, ticagrelor or prasugrel.

^d^ At least 5L/min.

### In-hospital course and predictors of 30-day mortality

**Figure 1:**
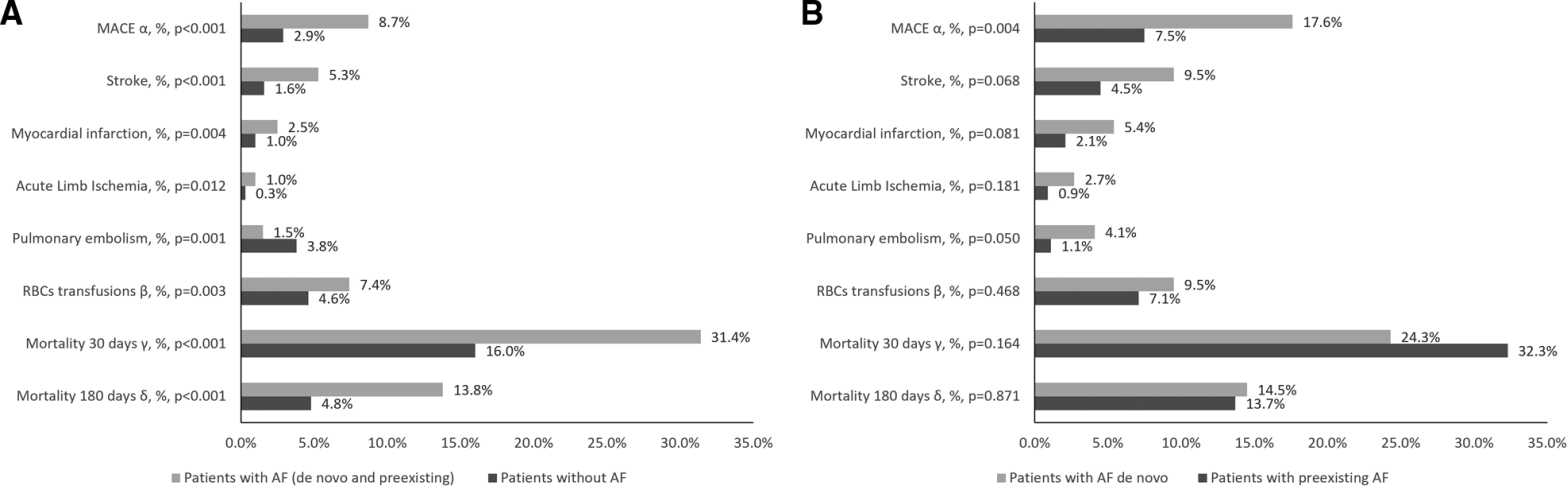
Outcomes among patients without AF and with AF (**A**) and among patients with pre-existing AF and with AF *de novo* (**B**). Data are presented as %. *p* < 0.01 for between AF group and No AF group difference; *^α^* –MACE, major adverse cardiovascular events including any incident stroke, myocardial infarction or acute peripheral artery ischemia; *^β^* - need for red blood cells transfusion was used as a surrogate for major bleeding events; *^γ^* - death from any cause in 30-days follow up since hospital admission; *^δ^* - death from any cause in 180-days follow up after hospital discharge; (analysis for patients discharged alive from hospital: 3,641 patients without AF and 419 patients with AF); abbreviations: AF, atrial fibrillation; MACE, major adverse cardiovascular event.

**Figure 2:**
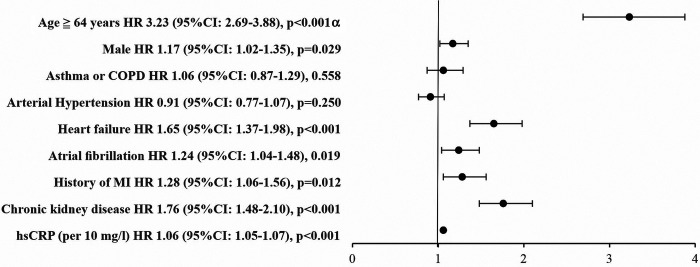
Cox regression analysis: independent predictors of 30-day mortality (whole study group; *N* = 4998). *^α^* - median for age in whole study group was 64 years; abbreviations: CI, confidence interval; COPD, chronic obstructive pulmonary disease; HR, hazard ratio; hsCRP, high-sensitivity C reactive protein; MI, myocardial infarction.

Patients with AF in comparison to patients without AF had a higher frequency of MACEs and need for RBCs transfusion, while the event rate for pulmonary embolism was lower in this group. The short-term mortality among AF patients was significantly higher than in the group without AF (31.4% vs. 17.0%, *p* < 0.001, Figure 1A). The frequency rate of MACEs was significantly higher in patients with AF *de novo* in comparison to those with preexisting AF (Figure 1B). In the whole study group, in multivariate Cox regression analysis, AF was an independent predictor of short-term mortality (HR: 1.236, 95% CI: 1.035–1.476) along with age ≥64 years, hsCRP, heart failure, diabetes, chronic kidney disease or history of MI (Figure 2).

### Sub-analyses of patients with atrial fibrillation

In the entire AF group of patients (*N* = 609), the median CHA_2_DS_2_-VASc score was 2.0 [IQR: 1.0–3.0]. For further analysis we excluded from this group patients with clinical conditions which could have an impact on the selection of anticoagulation strategy, as described above. Consequently, 421 AF patients (median [IQR] age 76.0 [70.0–83.0] years) remained in this sub-analysis. Table 3 shows the frequency of individual anticoagulants use by the CHA_2_DS_2_-VASc categories in this analysis. In the subgroup of patients who were eligible for anticoagulation treatment (CHA_2_DS_2_-VASc > 1 in men or >2 in women; *n* = 229) a higher number of deaths at 30 days follow up (59/68.6% vs. 27/31.4%; *p* < 0.001) was observed in patients without any anticoagulation treatment in comparison to those treated with any anticoagulation (Supplementary Table S1).

**Table 3:** Anticoagulation treatment in AF patients according to CHA_2_DS_2_-VASc score; *n* = 421^a^.

CHA_2_DS_2_-VASc, no. (%)	0 in men and ≤1 in women 64 (15.2)	1 in men or 2 in women 128 (30.4)	>1 in men or >2 in women 229 (54.4)
**Anticoagulation treatment**			
Any anticoagulation treatment, no. (%)	34 (53.1)	74 (57.8)	114 (49.8)
NOAC, no. (%)	19 (29.7)	29 (22.7)	57 (24.9)
VKA, no. (%)	2 (3.1)	5 (3.9)	5 (2.2)
LMWH, no. (%)	13 (20.3)	40 (31.3)	52 (22.7)
No anticoagulation treatment, no. (%)	30 (46.9)	54 (42.2)	115 (50.2)

LMWH, low molecular weight heparin; NOAC, novel oral anticoagulants; VKA, vitamin K antagonists.

^a^ Patients with requirement for mechanical ventilation, renal replacement therapy or those who were treated during hospital stay by two different anticoagulants or were switched within any anticoagulant agent to another (VKA vs. NOACs vs. LMWH) were excluded;.

**Figure 3:**
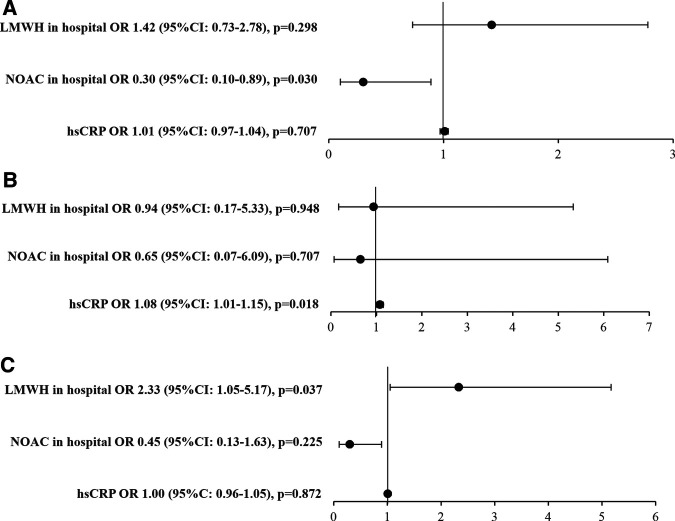
Multivariable logistic regression, *n* = 421*. Predictors of: (**A**). MACE *^α^*, (**B**). Pulmonary embolism, (**C**). RBCs transfusions *^β^*. *^α^* - MACE, major adverse cardiovascular events including any incident stroke, myocardial infarction or acute peripheral artery ischemia; *^β^* - need for red blood cells (RBCs) transfusion as a surrogate for major bleeding events; * - patients with requirement for mechanical ventilation, renal replacement therapy or those who were treated during hospital stay by two different anticoagulants or were switched within any anticoagulant agent to another (VKA vs. NOACs vs. LMWH) were excluded; abbreviations: CI, confidence interval; hsCRP, high-sensitivity C reactive protein (increase per 10 mg/L); LMWH, low molecular weight heparin; NOAC, novel oral anticoagulants; OR, odds ratio; RBCs, packed red blood cells.

In the group of 421 patients with AF, independent predictor of lower risk of MACE was the use of NOAC in hospital (Figure 3, Panel A). The risk of pulmonary embolism was related only to increased hsCRP level with no influence of anticoagulation use (Figure 3, Panel B). The need for RBCs transfusion was higher among AF patients treated with LMWH as compared to treated with NOACs (Figure 3, Panel C).

**Figure 4:**
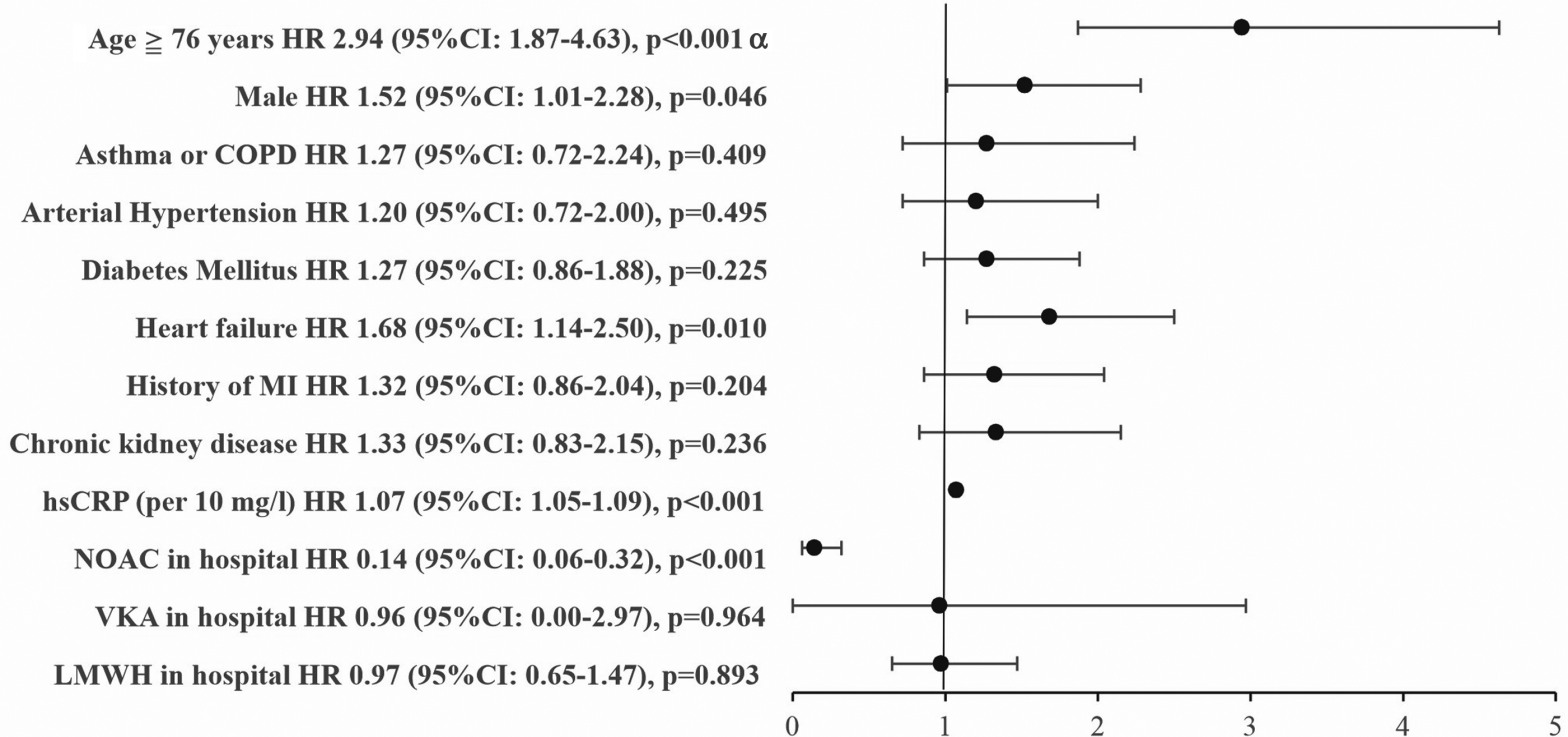
Cox regression analysis: independent predictors of 30-day mortality in AF patients, *n* = 421*. * - patients with requirement for mechanical ventilation, renal replacement therapy or those who were treated during hospital stay by two different anticoagulants or were switched within any anticoagulant agent to another (VKA vs. NOACs vs. LMWH) were excluded; *^α^* - median for age in AF patients (*n* = 421) was 76 years. Abbreviations: CI, confidence interval; COPD, chronic obstructive pulmonary disease; HR, hazard ratio; hsCRP, high-sensitivity C reactive protein; LMWH, low molecular weight heparin; NOAC, novel oral anticoagulants; VKA, vitamin K antagonists.

In Cox-proportional hazard model analysis: heart failure, age ≥76 years and hsCRP were associated with an increased risk of 30-day mortality, whereas the use of NOACs was associated with a reduced risk of 30-day mortality (Figure 4).

### Long-term post-discharged survival analysis

**Figure 5:**
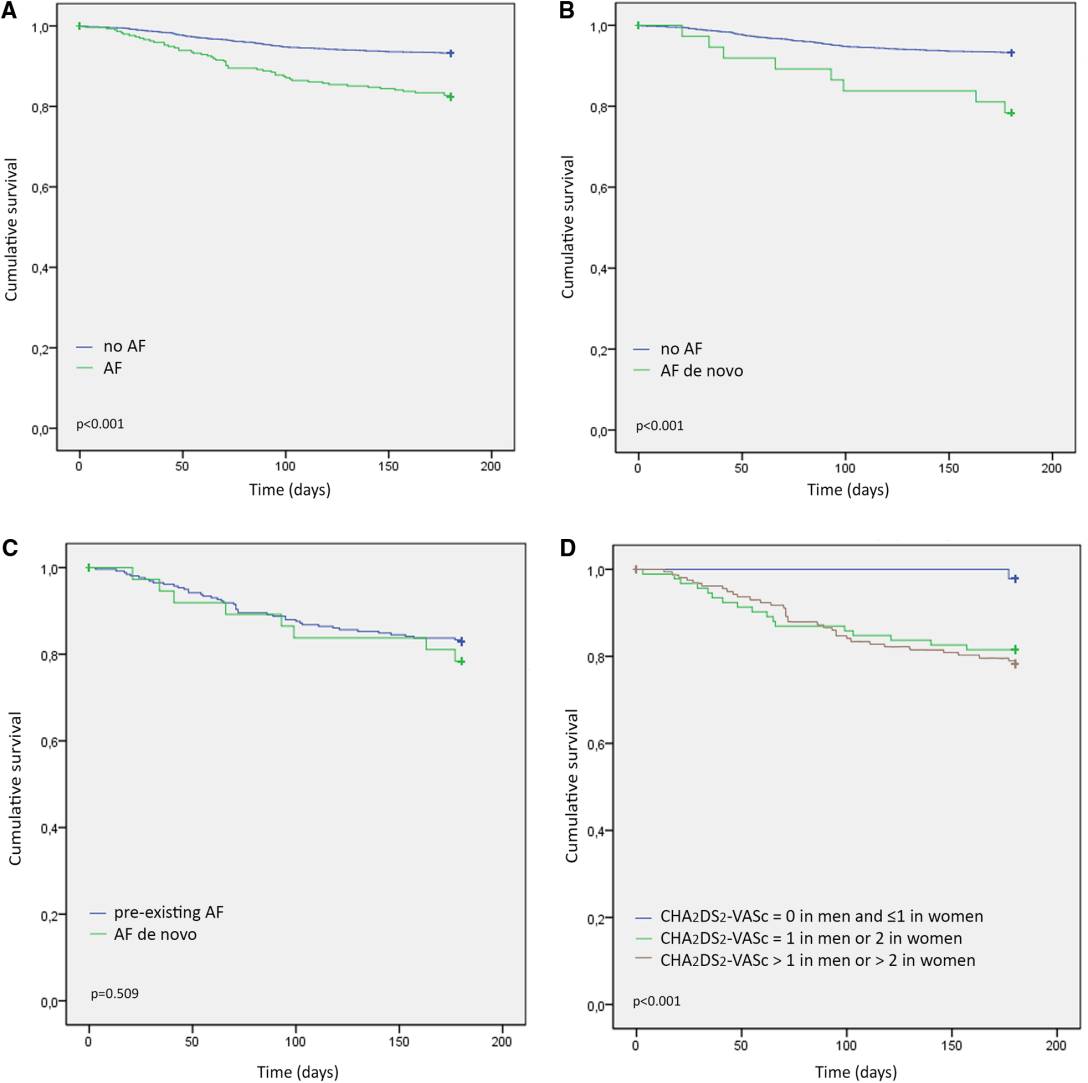
Kaplan–meier 180-day post-discharge survival probability stratified by: (**A**) the presence of AF, (**B**) AF *de novo* vs. without AF, (**C**). AF *de novo* vs. pre-existing AF, (**D**). CHA_2_DS_2_-VASc score: Group 1: CHA_2_DS_2_-VASc = 0 in men and ≤1 in women; Group 2: CHA_2_DS_2_-VASc = 1 in men or 2 in women; and Group 3: CHA_2_DS_2_-VASc > 1 in men or >2 in women. Abbreviations: AF, atrial fibrillation.

Data regarding the death from any cause at the 180-day after hospital discharge were available for 4,060 patients (i.e., 100.0% of patients discharged alive from hospital) including 419 patients with AF (Supplementary Table 1). Kaplan-Meier estimates showed that the survival probability of patients with AF was lower than in patients without AF. Similar results were observed when comparing *de novo* AF patients against the group without pre-existing AF. Thus, the survival probability was similar in patients with either pre-existing or *de novo* AF. Higher CHA_2_DS_2_-VASc scores was associated with a higher mortality at the 180-day endpoint (Figure 5).

## Discussion

Our findings confirm that AF is an important prognostic factor for short-term mortality outcome in patients hospitalized due to COVID-19. Moreover, the use of NOACs in patients with AF was associated with reduced short-term mortality in the 30-day observational period. We also showed that COVID-19 patients with either pre-existing or *de novo* AF at hospitalization have higher risk of long-term mortality after 180-days of follow up period after hospital-discharge as compared to patients without AF.

The prevalence of AF among COVID-19 patients in other studies ranged between 3.5%–15% (11, 12, 16–19, 35, 36), however, in patients with cardiac comorbidities AF was observed in approximately 35% (10) and among patients who had died due to COVID-19 the prevalence of AF reached almost 40% in one report (13). Previous analyses that focused on *de novo* cases of AF during hospitalization in patients with COVID-19 indicated that the prevalence of *de novo* AF varied from 5% to 15% depending on age, clinical severity of COVID-19 and the number of co-morbidities (17, 35–39). In our study, overall, 12.2% of hospitalized patients with COVID-19 had AF and only 1.5% had *de novo* AF. The latter result is in agreement with results obtained in a large meta-analysis of Zuin et al. where pre-existing AF was observed in 11% of COVID-19 patients (19). The large discrepancy between different studies in diagnosed *de novo* AF may be due to a difference in definitions of *de novo* AF (i.e., AF regardless of previous AF diagnosis or the first documented AF episode in a lifetime) (19).

AF has been already previously linked to infectious conditions including acute respiratory infections, sepsis or other critical illnesses (40–43). Not surprisingly, acute respiratory infections in patients with COVID-19 may also represent a precipitating factor for new-onset or recurrent AF. COVID-19 associates with inflammation and cytokine activation and possibly with direct myocardial injury (3, 4). Moreover, during acute COVID-19, additional factors including electrolyte abnormalities, dehydration, hypoxia, and over-activation of the sympathetic nervous system may contribute to the development of arrhythmias including AF (6–9, 14). Based on our data, it seems that in patients with AF, factors such as age and cardiac comorbidities may be associated with more severe inflammation (higher procalcitonin and IL-6 levels in AF group in comparison to non-AF group) and more pronounced cardiac involvement (higher NT-pro-BNP and hs cTnI levels) in the AF group in comparison to group without AF. We suggest that the described above factors may contribute to the observed higher 30-day mortality rate in the AF group in comparison to patients without AF.

The impact of AF on short-term mortality in patients with COVID-19 was described in previous studies. Peltzer et al. found that the presence of AF or atrial flutter was independently associated with 30-day mortality (Odds Ratio: 1.93) (16). In the Cardio-COVID Italy multicentre study (*n* = 696 patients), COVID-19 severity and cardiovascular comorbidities increased the risk of in-hospital mortality (HR: 1.73) in AF patients, after adjustment for clinical confounders. This HR is slightly higher than in or study which may be explained by the older age in the Italian cohort.

In our registry, the use of anticoagulation treatment in patients with AF was lower than could be expected according to current ESC recommendations for the management of AF (2018) (10). The ESC COVID-19 guidelines published in 2,022 maintained the same recommendations for the use of anticoagulation treatment in COVID-19 patients with AF due to insufficient evidence for a different anticoagulation strategy in these patients (7). The rate of AF patients with inappropriate anticoagulation strategy in our registry was similar to those reported by other investigators during the COVID-19 pandemic (44, 45). One of the causes might be the fact that treatment strategies have markedly varied over time in patients with AF complicated by COVID-19 (20, 21, 26–28).

An increased risk for MACEs and thrombotic events is a typical complication for patients with AF (10), which explains the higher incidence of in-hospital MACEs in patients with AF compared to patients without AF also in our study. A lower rate of symptomatic venous embolic events as assessed by pulmonary embolism events in our study, among patients with AF as compared to patients without AF is likely attributable to the use of anticoagulants in AF patients (46). In previous studies, the risk for pulmonary embolism was related to inflammation severity (47, 48), however, in our study the group with AF and without AF did not differ in inflammation severity assessed by hsCRP level.

Our comparative results of the subgroup of patients with *de novo* vs. pre-existing AF regarding the rate of MACE requires a separate comment. In our study, we found that although there were no significant differences in the rate of individual events evaluated separately, (i.e., myocardial infarction, stroke, acute peripheral artery ischemia), the composite end-point occurred significantly more often in the subgroup of patients with *de novo* AF compared to patients with pre-existing AF. Similar trend was observed by us for the frequency rate of pulmonary embolism. The above indicates that a recorded first-time episode of AF triggered by pulmonary infection cannot be viewed as benign, transient, or insignificant. The results of other investigators also indicate that patients with *de novo* AF discovered during a pulmonary infection, especially in the absence of previously effective anticoagulation, have an increased risk of adverse events and require special attention (49).

On the basis of our work, it is also worth considering whether the correlations we have shown regarding the relationship between atrial fibrillation and the clinical course during SARS-CoV2 infection are not also universal for other pathogens (viral or bacterial) leading to pneumonia, sepsis or septic shock. Based on an analysis of the available literature, it should be noted that the relationship between AF and pneumonia had been well described in pre-COVID-19 era (49, 50), however there is a lack of extensive comparative analyses of the association between AF and pneumonia depending on the pathogen causing it (SARS-CoV2 vs. other viral or bacterial infections). Comparative data on differences in the incidence of thromboembolic events in patients with SARS-CoV2-induced pneumonia compared to other pathogens responsible for pneumonia are also scarce (51). Instead, we can assume that there are similar pathophysiological mechanisms leading to an increased incidence of AF (particularly AF *de novo*) comorbidities and thromboembolic complications among patients with infection giving clinical manifestation of pneumonia regardless of the causative agent (i.e., systemic inflammation, metabolic imbalances or hypoxia, leading to cardiac injury as well as endothelial cell dysfunction secondary to infection, etc) (3, 4, 52).

It is also important emphasizing that there are some pathophysiological explanations indicating that patients who develop AF *de novo* during pneumonia have increased risk of bleeding events (depletion of coagulation factors and platelets as a result of inflammatory response during infection) (53). Based on the available data, it can be conservatively assumed that among patients with SARS-CoV2 infection, the risk of both thromboembolic and hemorrhagic complications to which AF patients are particularly vulnerable is at least comparable to that of patients with pulmonary infection caused by other pathogens (bacterial or viral) (51, 53, 54). Considering the rationale presented above, it is worth emphasizing that the association between AF and pneumonia is not limited only to the COVID-19 pandemic, and the results we obtained may provide a point of consideration for patients with community acquired pneumonia caused by other causes than SARS-CoV2 virus infection.

In our study, NOAC use in AF patients was associated with a lower risk for MACEs in contrast to LMWH. Importantly, although detailed data on bleeding events were not available and thus analyzed in our study, the use of NOACs in AF patients was not associated with an increased need for RBCs transfusions. Moreover, we found that the use of NOAC in AF patients was associated with a significant reduction of short-term mortality. In the CORIST Study beneficial effects of NOAC treatment in patients with AF admitted due to COVID-19 were also observed but did not reach statistical significance, probably due to low number of patients with AF (*n* = 154, 3.5%) (18). Denas et al. (55) found in older adults with COVID-19 that individuals on chronic oral anticoagulant treatment (including VKA and NOAC) for AF had significantly lower all-cause mortality rate than non-anticoagulated patients (26.5% vs. 32.2%; *p* = 0.036). However, reduced mortality in response to anticoagulation was not significant in this study in time to event analysis (55). In our study, we confirmed a beneficial influence of NOAC treatment on survival among AF patients with COVID-19 while a corresponding analysis for VKA appears not meaningful due to the low number of patients that were treated with VKA.

To date, there are only a few studies that evaluated long-term follow-up in patients with COVID-19 with very limited data about concurrent cardiovascular comorbidities such as AF on post-discharge mortality (56–58). Our results clearly demonstrate that patients with AF have a worse long-term prognosis after hospital discharge compared to patients without AF. This observation applies to the whole AF group as well as to patients with *de novo* AF. The lack of difference in survival probability among patients with *de novo* and preexisting AF further confirms that *de novo* AF in patients with COVID-19 should not be considered as a transient phenomenon caused by COVID-19, but should be considered as condition that indicates a poorer prognosis in follow up. Our findings confirm that COVID-19 patients with AF require closer monitoring and optimal treatment after hospital discharge. The optimization of treatment strategies and development of additional prognostic tools may help reduce the risk of mortality. For example, the association between the CHA_2_DS_2_-VASc score and mortality has already been demonstrated in patients with COVID-19 (59). Based on our results, we have seen that CHA_2_DS_2_-VASc scoring can also be used as a risk stratification tool for mortality in long term follow-up.

### Study limitations

We acknowledge that there are several potential limitations to this study. Firstly, not all patients admitted to the hospital were monitored using continuous electrocardiographic monitoring and the diagnosis of AF was mainly carried out using ECG tracings. As a result, the occurrence of paroxysmal AF during hospitalization may have been underdiagnosed. It is also entirely possible that some interpreted as *de novo* cases of AF may in fact have been present prior to the COVID-19 infection but were not diagnosed earlier. Furthermore, due to the nature of the COVID-19 pandemic, specialists with limited cardiological training assumed the care of many of the patients admitted to the University Hospital, which may partially explain why there was a lower-than-expected prescription rate for antithrombotic prophylaxis in AF patients. Additionally, we should be cautious in drawing conclusions based on our sub-analysis of patients with AF and the use of OAC because a significant proportion of patients have been excluded from this analysis (i.e., patients with a clinical condition which could have an impact on the selection of anticoagulation strategy) which may have influenced some selection bias in this subgroup analysis. Another limitation is lack of information about anticoagulation treatment adherence in patients with AF after hospital discharge. We used the need for RBCs transfusion as a surrogate for bleeding outcomes which should be interpreted cautiously due to possible variations in transfusion strategies and the possibility that transfusions were given irrespective of a bleeding event.

In conclusion our findings expand previous evidence that among patients with chronic NCDs those with AF hospitalized due to COVID-19 have a poor prognosis. Both pre-existing and *de novo* AF is associated with increased mortality in short- and long-term follow-up. The use of NOACs exhibited a profound beneficial effect on outcomes including reduced mortality and MACE.

## Data Availability

The raw data supporting the conclusions of this article will be made available by the authors, without undue reservation.
