# Supplementary material for: Prevalence and clinical implications of atrial fibrillation in patients hospitalized due to COVID-19: Data from a registry in Poland
Source: Front Cardiovasc Med. 2023 Mar 13;10:1133373. doi: 10.3389/fcvm.2023.1133373 (PMC10041565; doi:10.3389/fcvm.2023.1133373)
Supplement: Supplementary file 2 [file Table1.docx]

Supplementary Table 1.

Basic characteristics of post-discharge survivors followed up for 180 days. (n=4060)

| Characteristics | No AF  N=3641  (89.7%) | AF  N= 419  (10.3%) | p-value ^$^ |
| --- | --- | --- | --- |
| Age, yrs, median (IQR) | 61 (47-70) | 74 (68-81) | <0.001 |
| Male sex, n (%) | 1963 (53.9) | 219 (52.3) | 0.278 |
| BMI*, kg/m2, mean (SD) | 29.02 (5.50) | 28.90 (5.60) | 0.638 |
| *Pre-Existing*  *conditions, n (%)* |  |  |  |
| Arterial hypertension | 1958 (53.8) | 347 (82.8) | <0.001 |
| Hyperlipidemia | 688 (18.9) | 153 (36.5) | <0.001 |
| Diabetes Mellitus | 843 (23.2) | 156 (37.2) | <0.001 |
| Coronary Artery Disease | 435 (11.9) | 145 (34.6) | <0.001 |
| History of MI | 224 (6.2) | 77 (18.4) | <0.001 |
| Heart Failure | 159 (4.4) | 131 (31.3) | <0.001 |
| Atrial Fibrillation de novo | 0.0 (0.0) | 55 (13.1) | <0.001 |
| History of Stroke | 211 (5.8) | 65 (15.5) | <0.001 |
| Asthma | 225 (6.2) | 27 (6.4) | 0.448 |
| COPD | 149 (4.1) | 34 (8.1) | <0.001 |
| Chronic kidney disease | 234 (6.4) | 52 (12.4) | <0.001 |

Abbreviations: AF, atrial fibrillation; BMI, body mass index; COPD, chronic obstructive pulmonary disease; MI, myocardial infarction.
